# A Machine Learning Method for Drug Combination Prediction

**DOI:** 10.3389/fgene.2020.01000

**Published:** 2020-08-25

**Authors:** Jiang Li, Xin-Yu Tong, Li-Da Zhu, Hong-Yu Zhang

**Affiliations:** Hubei Key Laboratory of Agricultural Bioinformatics, College of Informatics, Huazhong Agricultural University, Wuhan, China

**Keywords:** drug combination, multifeature, paclitaxel, neighbor recommender method, ensemble learning

## Abstract

Drug combination is now a hot research topic in the pharmaceutical industry, but experiment-based methodologies are extremely costly in time and money. Many computational methods have been proposed to address these problems by starting from existing drug combinations. However, in most cases, only molecular structure information is included, which covers too limited a set of drug characteristics to efficiently screen drug combinations. Here, we integrated similarity-based multifeature drug data to improve the prediction accuracy by using the neighbor recommender method combined with ensemble learning algorithms. By conducting feature assessment analysis, we selected the most useful drug features and achieved 0.964 AUC in the ensemble models. The comparison results showed that the ensemble models outperform traditional machine learning algorithms such as support vector machine (SVM), naïve Bayes (NB), and logistic regression (GLM). Furthermore, we predicted 7 candidate drug combinations for a specific drug, paclitaxel, and successfully verified that the two of the predicted combinations have promising effects.

## Introduction

With accumulating research in systematic pharmacology and clinical experiences, the “one drug, one target” therapeutic mode is found to be limited. The effects of single-target drugs on complex diseases are not satisfactory since complex diseases like cancer are usually regulated by numerous different genes and regulation pathways rather than by single genes. Drug combinations have been designed to achieve better efficacy and fewer side effects than each individual drug (Musa et al., 2018; Sheng et al., 2018). Traditionally, combinations of drugs tend to be discovered by biological experiments involving massive selection (Sheng et al., 2018). However, screening synergistic combinations by experimentation is costly and time consuming. Therefore, it is urgent to screen drug combinations efficiently and economically. The increasing experimental data from multiple sources, such as genetics, chemical structures and gene expression profiles, provide an excellent research foundation for computational methods to investigate drug combinations. Currently, many researchers focus on machine learning-based computational methods and biotext mining from electronic medical reports to accelerate drug combination identification.

With the development of the pharmaceutical industry and high-throughput screening of the human genome, large amounts of drug information are generated, and many clinical and drug databases are publicly available online. Databases including DrugBank (Law et al., 2014), the Therapeutic Target Database (TTD) (Chen et al., 2002) and the Drug Gene Interaction Database (DGIdb) (Wagner et al., 2016) contain experimentally proven drug-target and drug-indication information, which provide us with comprehensive multiomics drug information. Large numbers of drug gene expression profiles, such as the Connectivity Map (CMap) (Lamb et al., 2006), are accumulated because of the rapid development of high-throughput techniques. Other drug data, such as enzymes, side effects and pathways, are also obtainable in several databases (e.g., DrugBank, KEGG, and SIDER). In addition, existing drug combinations are collected in data portals such as the Drug Combination Database (DCDB) (Liu et al., 2014), which consists of 1363 pairs in total. These enriched supplies of drug information enable us to approach data-driven prediction problems such as drug combinations.

Recently, many new techniques and methods have been established based on the assumption that “similar drugs have similar activity” to predict the synergistic effects of drug combinations. These methods have tried to predict new drug combinations based on similarity to existing drug combinations (Chen et al., 2016). Cheng et al. (Cheng and Zhao, 2014) applied five kinds of algorithms [naïve Bayes, decision tree, k-nearest neighbor, logistic regression, and support vector machine (SVM)] using four similarity-based features; Vilar et al. (2013) proposed the chemical structure similarity-based prediction method and predicted a large number of new combinations; Zhang et al. (Zhang et al., 2017, 2019; Wen et al., 2018) used the neighbor recommender method, the random walk method and the matrix perturbation method to build prediction models, then they further explore matrix factorization method and ensemble method on this problem; Shi et al. (2018) developed a matrix factorization method with a DDI network and drug side effects vector feature to detect unknown drug combinations; and Lee et al. (2019) constructed a deep learning network with autoencoders to accurately find more drug combinations. These methods provide promising and applicable approaches to systematically detect unknown drug combinations with multifeature drug properties.

In this paper, we proposed a machine learning approach to predict potential drug combinations by integrating multiple drug features. Since the data sparsity of drug information is a key challenge in multiple feature prediction, the neighbor recommender method (NRM) (Zhang et al., 2017) was introduced to address this problem by leveraging the feature similarity rating matrix for drug pairs. First, we collected multiple features of drugs, including drug-indication data, drug-target data, drug-induced gene expression data, chemical structure information and known drug-drug combinations from different sources. Multisource data provided biological information, phenotypic information and known combinations to fully characterize drug-drug combinations. The Tanimoto coefficient was used to measure the similarity between drugs in terms of each feature. Then, three different classification models with downsampling methods were constructed, and the SVM model with the best performance was selected for later comparison. The drug combination prediction model was considered a similarity-based problem in many methods, since under the assumption that drugs with more similarities are likely to have similar functionality. To make use of diverse information, the neighbor recommender method was used to generate similarity-based models based on every selected feature of the drug. Finally, the ensemble model was built by combining multiple feature-based models as basic predictors using an ensemble learning algorithm. According to the performances of the prediction models, we evaluated the usefulness of different drug information sources for drug combination prediction. Afterward, the ensemble model and SVM classification model were compared, and the ensemble model was selected as the best prediction model. Furthermore, by comparison to several state-of-the-art algorithms, we achieved better performances with NRM and ensemble learning, and a maximum AUC value of 0.964 was obtained, which indicated the reliability and universality of our method. To further show this point, we used our method to predict drug combinations for a specific drug, paclitaxel, and obtained seven candidate drug combinations. We also successfully verified two predicted combinations that had promising effects.

## Materials and Methods

**FIGURE 1:**
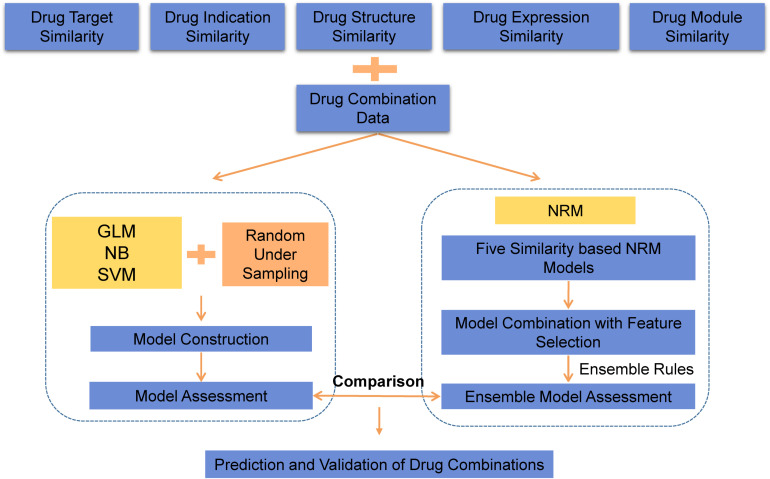
The workflow of drug combination prediction.

As an overview, a flowchart of our method is depicted in Figure 1. The primary processing consisted of several steps: (A) construct the similarity feature-based model according to the drug feature profiles; (B) select the useful features and construct the ensemble model for drug combination prediction; and (C) use the model to predict potential drug combinations and conduct experimental validation.

### Raw Dataset of Annotated Drug Combinations

Since our classification methods require existing drug combinations for training, the known drug combinations downloaded from the DCDB and PreDC databases (Li et al., 2015) were labeled positive samples. In our paper, the combination was performed on pair-wise drugs, and some drug combinations consist of more than two chemical compounds, we chose every two drugs in those combinations and marked them as a pair.

### Multifeature Information

In this paper, we considered five drug features to characterize the similarity of drug pairs, including drug-indication data, drug-target data, drug-induced gene expression data, and chemical structure information.

Drug indication information was derived from SCG-Drug (Quan et al., 2019), while the disease names were previously unified by using UMLS (similarity threshold 0.75). Drug instances and targets were mainly collected from DrugBank, TTD and DGIdb. Drug gene expression profiles were provided by CMap (Lamb et al., 2006) as an important drug feature (Musa et al., 2018). The chemical structural similarity of drug pairs was calculated by the online tool PubChem (Kim et al., 2019) with the substructure key-based 2D Tanimoto similarity score of each drug pair (scores ranging from 0 to 100). The drug module information on indications was calculated using a bioclustering method based on drug expression profile data (Xiong et al., 2014). Finally, we obtained 606 drugs with multifeature information.

### Drug Similarity Measurement

With large amounts of data generated, a drug could be represented by a fingerprint vector in different data types. To gain the best model performance, a suitable similarity measure was needed as important prior knowledge.

The Tanimoto coefficient (also known as the Jaccard coefficient) is used to determine the similarity between drug pairs. The Tanimoto coefficient score is calculated using the following equation:

$$\mathrm{Tanimoto}\,\mathrm{coefficient}=\frac{N_{\text{AB}}}{N_{\text{A}}+N_{\text{B}}-N_{\text{AB}}}$$

Where *N*_*A*_ is the number of drug A-related targets, indications, modules, genes and structures; *N*_*B*_ is the number of drug B-related targets, indications, modules, genes and structures, and *N*_*AB*_ is the number of common targets, indications, modules, genes and structures for drug A and drug B. The value of the Tanimoto coefficient ranges from 0 to 1.

### Classification Method for Drug Combination Prediction

#### Logistic Regression

Logistic regression (GLM) (Cheng and Zhao, 2014) is used to estimate the probability of the response variable using a logistic function. In our data, the output of the GLM model consists the probabilities of “existing combinations” and “noncombinations.”

#### Naïve Bayes

Naïve Bayes (NB) (Watson, 2008) is the simplified version of the Bayesian method, which is based on the hypothesis that each attribute is independent. The NB algorithm calculates the posterior probability of an instance by the following equation:

$$P\,\left(B|A\right)=\frac{P\,\left(A|B\right)\,P\,\left(B\right)}{P\,\left(A\right)}$$

#### Support Vector Machine

The SVM (Cheng and Zhao, 2014) is a powerful method for classification. It separates the dataset by maximizing geometric spacing and mapping data points into a high-dimensional space. Kernel parameter γ and penalty parameter C are useful when searching the optimal SVM model.

In terms of our data, every drug pair was represented by a vector of five dimensions (using Tanimoto coefficients extracted from five drug features) along with one category label.

#### The Neighbor Recommender Method for Drug Combination Prediction

The neighbor recommender method (NRM) is widely deployed in industry. We had multisource data that provide diverse information and confirmed drug combinations. Here, we applied NRM to those multisource data and predicted the drug combination. We calculated the probability of two drugs with the following equation (Zhang et al., 2017):

$$Y_{i\,j}=\sum_{k=1,k\neq j}^{N}S_{i\,k}\,a_{k\,j}/\sum_{k=1,k\neq j}^{N}S_{i\,k}$$

where N is the number of drugs, when calculating the possibility between drug_*i*_ and drug_*j*_, *S*_*ik*_ indicates the similarity between drug_*i*_ and other drugs in the similarity matrix (except drug_*j*_), and *a*_*kj*_ is 1 or 0, which represents whether there is interaction or noninteraction between drug*_*j*_* and drug*_*k*_*. The probability of drug*_*i*_* interacting with drug*_*j*_*, score*_*ji*_* = score*_*ij*_* = *Y*_*ij*_ + *Y*_*ji*_.*Y_*ji*_*, is calculated in the same way as *Y*_*ij*_.

#### Ensemble Learning for Drug Combination Prediction

Since we used the NRM method to generate models based on five features, it was natural that we adopted ensemble rules to obtain better model performances. To the best of our knowledge, the two most commonly used ensemble rules are the weighted average ensemble and classifier ensemble rules (Zhang et al., 2017). We adopted GLM classifier rules to finalize the output from the base predictors.

### The Selection of Drug Features

In total, we collected five drug features to build drug combination prediction models. However, not every feature was necessary to include. Here, we implemented ensemble learning on different numbers of features to find the most relevant ones.

Since we used NRM to generate five different models, we first used all outputs of the five models as basic predictors in the ensemble method. This model was considered the benchmark output. Then, we sorted the models in reverse order based on model performance. Finally, we combined different outputs of NRM models to fit ensemble models in turn. Comparison with the benchmark model was conducted to select the most relevant drug features.

NRM is based on the hypothesis that drugs with high similarity tend to have similar activity. We deployed two significant difference tests, the Kolmogorov-Smirnov test (KS-test) and Student’s test (*T*-test), to analyze the feasibility of five drug features by comparing the difference in Tanimoto values between positive and negative samples. In the KS test, the value of D, which represents the maximum vertical difference between two cumulative distribution curves, was extracted to evaluate the difference between positive and negative classes. The range of D is from −1 to 1.

### Evaluation Metrics

We used *k*-fold cross validation to evaluate the models, and the value of *k* was within 3, 5, and 10. Since the sampling method was taken into consideration, we repeated the sampling process 1000 times to prevent data bias, and the average performances were the final result.

Two metrics for common binary classification problems, the area under the ROC curve (AUROC) and the area under the precision-recall curve (AUPR), were used to evaluate the models regardless of the threshold. Other machine learning metrics were also used: f1 score, recall and precision. These three metrics were calculated from the number of true positives (TP), false positives (FP), true negatives (TN), and false negatives (FN) using the following equations:

$$\text{Recall}=\frac{\text{TP}}{\text{TP}+\text{FN}}$$

$$\text{Precision}=\frac{\text{TP}}{\text{TP}+\text{FP}}$$

$$F\,1\,\text{score}=2\times \frac{\text{Precision}\times \text{Recall}}{\text{Precision}+\text{Recall}}$$

### Experiments on Drug Combinations

#### Cell Culture and Reagents

A375 (human melanoma cell line) was purchased from Procell. Testing drugs, including paclitaxel and monobenzone, were purchased from Selleck. Medium and other chemicals used in cell culture were purchased from MedChemexpress. Cell Counting Kit-8 was purchased from Bimake. A microplate spectrophotometer (EON) was purchased from BioTek.

#### Growth Inhibition Assay *in vitro*

A375 cells were cultured overnight in Dulbecco’s modified Eagle’s medium (DMEM) supplemented with 10% fetal bovine serum (FBS) in a humidified atmosphere of 5% CO_2_ and 95% air at 37°C, then seeded in 96-well plates and incubated overnight. Next, A375 cells were incubated with different concentrations of the tested drugs or solvent control for 24 h. The tested drugs were diluted with 1% DMSO. After the drug treatment, the cell viability was measured using Cell Counting Kit-8 following the manufacturer’s instructions, and the absorbance at 450 nm for samples was measured using a microplate spectrophotometer. The half maximal inhibitory concentration (IC_50_) value of each drug was calculated using GraphPad Prism 7.0^1^. Each drug was tested in a concentration gradient, and the experiments were repeated in three biological replicates.

#### Combination Index Assay

Based on the IC_50_ values of paclitaxel and monobenzone monotherapy on A375 cells, nine drug combinations with different drug concentrations were determined to calculate the combination index (CI), a median effect principle proposed by Chou (2006). After the drug treatment, the cell viability was measured using a Cell Counting Kit-8 according to the manufacturer’s instructions, and the absorbance at 450 nm for samples was measured by using a microplate spectrophotometer and taking the average. The inhibition rates of the cells compared with the DMSO control group were calculated separately. All experiments were performed in triplicate. The combination index (CI) was calculated by CompuSyn software^2^. CI < 1, CI = 1, and CI > 1 represent synergism, additive effect and antagonism, respectively.

All machine learning algorithms were performed by using R (version 3.4.1). The R package e1071 was loaded for SVM and NB functions; the random forest algorithm was implemented using the R package randomForest; and GLM, *T*-test and KS-test were conducted with built-in functions of R. A *P*-value < 0.05 was considered statistically significant.

## Results

### Benchmarks

Due to the lack of existing gold standard datasets of known drug combinations, we annotated experimentally validated drug combinations from the DCDB and PreDC databases as benchmarks. Each annotation is curated and contains referenced information about the drug, including drug-indication data, drug-target data, drug-induced gene expression data, drug module data and chemical structure information. The benchmark includes only experimentally verified drug combinations.

### Impacts of Negative Sample Ratio Levels on the Prediction Performance

A total of 1,196 clinically validated drug combinations between 606 drugs were included in this work as positive samples. The remaining 182,119 samples $\left(C_{606}^{2}-1196\right)$ were considered negative samples, approximately 153 times the number of positive samples. Thus, our dataset was extremely unbalanced, which might lead to an overfitting problem. Since the positive data were far fewer than the negative data, we adopted a random downsampling method to regenerate negative samples to fit a model (Rayhan et al., 2017). In this research, we investigated how the performance varies when the ratio of negative samples to positive samples increases from 1 to 12. Three classical classifiers, including GLM, SVM, and NB, were employed for training and prediction.

### Performance of Random Sampling Classification Models

We used three machine learning algorithms under different ratios of positive/negative data to predict drug combinations (Figure 1).

**FIGURE 2:**
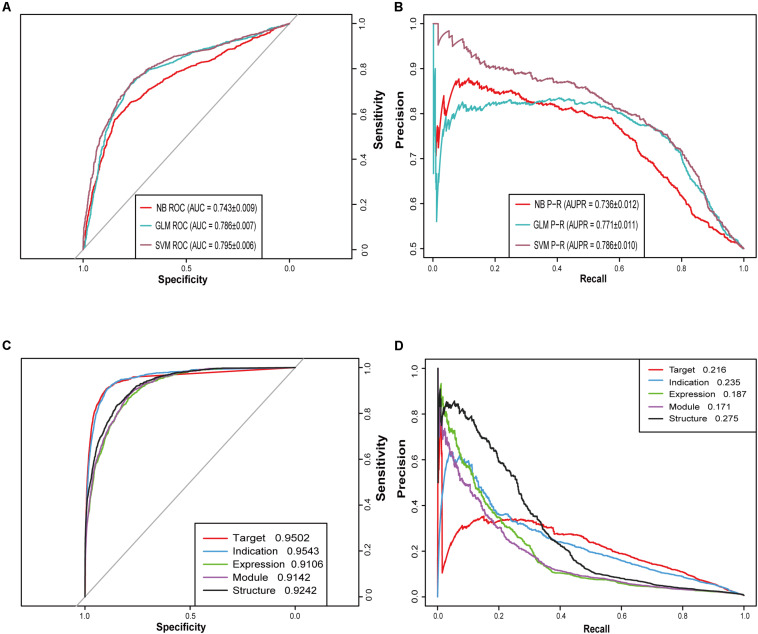
Receiver operating characteristic (ROC) curves and precision-recall (PR) curves for different models. **(A)** ROC curves of three machine learning algorithms (SVM, GLM, and NB) when the ratio of positive and negative samples is 1:1. **(B)** PR curves of three machine learning algorithms (SVM, GLM, and NB) when the ratio of positive and negative samples is 1:1. **(C)** ROC curves of five similarity-based NRM models. **(D)** PR curves of five similarity-based NRM models.

When the ratio of positive to negative samples was set to 1, the AUROC ranged from 0.743 to 0.795 (Figure 2A), and the AUPR ranged from 0.736 to 0.786 (Figure 2B). As shown in Table 1, the AUROC of the GLM and NB models was not sharply affected by the ratio of positive to negative classes, which was approximately 0.75. The values of the other four metrics declined significantly under different ratios of positive to negative classes. The performance of SVM was particularly heavily affected in terms of the AUROC value and all other metrics (Supplementary Table S1).

**TABLE 1:** Performances of different models with a sample ratio of positive: negative = 1: 1.

Model	k-Folds	Recall	AUROC	Precision	AUPR	F1 score
SVM	3	0.681 ± 0.005	0.793 ± 0.007	0.768 ± 0.009	0.782 ± 0.010	0.722 ± 0.006
	5	0.684 ± 0.005	0.795 ± 0.006	0.770 ± 0.009	0.785 ± 0.010	0.724 ± 0.006
	10	0.686 ± 0.005	0.795 ± 0.006	0.769 ± 0.009	0.786 ± 0.010	0.724 ± 0.006
NB	3	0.389 ± 0.018	0.742 ± 0.009	0.820 ± 0.015	0.733 ± 0.013	0.527 ± 0.018
	5	0.388 ± 0.018	0.742 ± 0.009	0.821 ± 0.013	0.734 ± 0.012	0.526 ± 0.018
	10	0.388 ± 0.018	0.743 ± 0.009	0.822 ± 0.014	0.736 ± 0.012	0.526 ± 0.018
GLM	3	0.598 ± 0.009	0.784 ± 0.007	0.805 ± 0.010	0.768 ± 0.011	0.686 ± 0.008
	5	0.599 ± 0.009	0.786 ± 0.007	0.806 ± 0.009	0.769 ± 0.011	0.687 ± 0.008
	10	0.599 ± 0.009	0.786 ± 0.007	0.806 ± 0.010	0.771 ± 0.011	0.686 ± 0.008
						

### Feature Analysis and Selection

To choose the most suitable features, we employed two feature analyses, the *T*-test and the KS-test. In the *T*-test, the median value of positive samples was significantly higher than that of negative samples in the features of drug-target similarity, drug-indication similarity and drug-structure similarity (*P* < 2E-16, *P* < 2E-16, and *P* = 0.012, respectively). The difference in the drug expression similarity feature was marginally significant (*P* = 0.1) (Supplementary Figure S1A). In the KS test, the overall value of positive samples was significantly higher than that of negative samples in the drug target similarity, drug indication similarity and drug structure similarity features (*P* < 2.2E-16, *P* < 2.2E-16, and *P* = 1.81E-03, respectively) (Supplementary Figure S1B).

The results of the feature analyses indicated that the features drug-target similarity, drug-indication similarity, drug-structure similarity and drug expression similarity tend to produce better performance.

Then, we built five NRM models based on different drug features to test the performance of each feature-based model and drug pair with prediction probability greater than or equal to 0.5 was considered positive. The performance of each NRM model is shown in Figures 2C,D according to the AUROC and AUPR curves: drug indication NRM model (DIM), drug target NRM model (DTM), drug structure NRM model (DSM), drug expression NRM model (DEM) and drug module NRM model (DMM). Detailed performances of the five NRM models in terms of recall, precision and f1 score are shown in Supplementary Table S4.

We further analyzed the importance of all features by using the random forest algorithm to quantify the feature importance measure by calculating the five feature-based models and providing the MeanDecreases Gini index. Then, the value of *MeanDecreaseGini* was normalized in the range 0–1 by using the min-max normalization method with the following equation:

$$\text{x}=\frac{\text{x}-m\,i\,n\,\left(x\right)}{m\,a\,x\,\left(x\right)-m\,i\,n\,\left(x\right)}$$

In Supplementary Figure S1C, the drug-target similarity showed the highest importance value, and the drug-module similarity had the lowest weight value. This result was consistent with the results of the previous feature analysis by *T*-test. These NRM models were used as base predictors to train ensemble models with selective combinations. In this paper, according to the feature analysis, we finally chose DTM, DIM, DSM and DEM to construct the final ensemble model.

### Performances of Ensemble Models With Feature Selection

The ensemble model is used here to combine all the suitable features from feature selection to achieve better performance. We applied a GLM classifier to integrate all five base predictors with the default parameters.

We investigated the ensemble classifier performances of different base predictor combinations based on the previous feature selection analysis and feature importance evaluation. The performance comparison in Table 2 shows that the ensemble classifier with four selective base predictors (DTM+DIM+DSM+DEM) outperformed the combination of all five predictors. This indicated that more features did not guarantee better performance.

**TABLE 2:** Performances of ensemble models.

Combination	K-Folds	Recall	AUROC	Precision	AUPR	F1 score
DTM+DIM+DSM+DEM	3	0.262 ± 0.021	0.957 ± 0.005	0.664 ± 0.020	0.383 ± 0.007	0.375 ± 0.025
	5	0.260 ± 0.054	0.957 ± 0.010	0.664 ± 0.042	0.383 ± 0.052	0.370 ± 0.053
	10	0.260 ± 0.051	0.957 ± 0.005	0.664 ± 0.090	0.384 ± 0.059	0.372 ± 0.062
All five basic models	3	0.260 ± 0.018	0.957 ± 0.005	0.654 ± 0.008	0.385 ± 0.009	0.372 ± 0.020
	5	0.257 ± 0.058	0.957 ± 0.010	0.650 ± 0.041	0.385 ± 0.050	0.365 ± 0.058
	10	0.256 ± 0.051	0.957 ± 0.006	0.642 ± 0.068	0.385 ± 0.061	0.364 ± 0.060
						

The selective feature-based ensemble classifier also outperformed the unbalanced dataset-trained SVM models, so the ensemble classifier was adopted as the best model for further research (Table 2).

### Comparison With Known State-of-the-Art Methods

We compared the model performances by implementing our ensemble method on three other published datasets. Yu (Yu et al., 2018) proposed a novel model for the prediction of drug combinations based on semi-nonnegative matrix factorization (DDINMF) with drug structure and off-label side effect information. Cheng (Cheng and Zhao, 2014) used four drug features with different machine learning methods (HNAI) to detect unknown drug combinations. Zhang (Zhang et al., 2015) adopted the label propagation algorithm (LPA) to predict unknown drug combinations. The dataset of each publication was downloaded according to the details in the papers, and five-fold cross validation was conducted on all datasets. All datasets were deployed with our proposed ensemble algorithm.

As shown in Table 3, our method outperformed LPA and HNAI. However, the performance was slightly lower than that of DDINMF. This result shows that our method has comparable performance to that of the state-of-the-art methods. More results of the performance comparison are shown in Supplementary Table S5.

**TABLE 3:** Comparison with state-of-the-art methods evaluated by five-fold validation.

Method	AUROC	AUPR	Method	AUROC	AUPR	Method	AUROC
DDINMF	0.872	0.605	LPA	0.926	0.729	HNAI	0.666
Our method	0.851	0.555	Our method	0.945	0.914	Our method	0.964

### Validation of Predicted Drug Combinations

In this research, we used our model to predict pairwise combination drugs for paclitaxel, which is an FDA-approved anticancer drug (Weaver, 2014). We split the dataset into training and test datasets. The training dataset represented drug pairs without paclitaxel, and the test dataset was the opposite. A drug pair in the test dataset with a probability greater than or equal to 0.5 was considered a potential combination of drugs related to paclitaxel.

Seven positive drug pairs were predicted using our best ensemble model. A large fraction of the newly predicted drug combinations (5 out of 7) were confirmed in the DCDB, and one drug combination (paclitaxel and camptothecin) was validated in the latest DrugBank database. The only undetected drug pair left for further study was paclitaxel and monobenzone (Table 4).

**TABLE 4:** Drug combinations predicted by the ensemble model.

Rank	Drug 1	Drug 2	Possibility
1	Monobenzone	Paclitaxel	0.885637514
2	Doxorubicin	Paclitaxel	0.876278314
3	Dexamethasone	Paclitaxel	0.833641682
4	Hydrocortisone	Paclitaxel	0.62772162
5	Paclitaxel	Prednisolone	0.606556951
6	Betamethasone	Paclitaxel	0.526487091
7	Camptothecin	Paclitaxel	0.525680295

**FIGURE 3:**
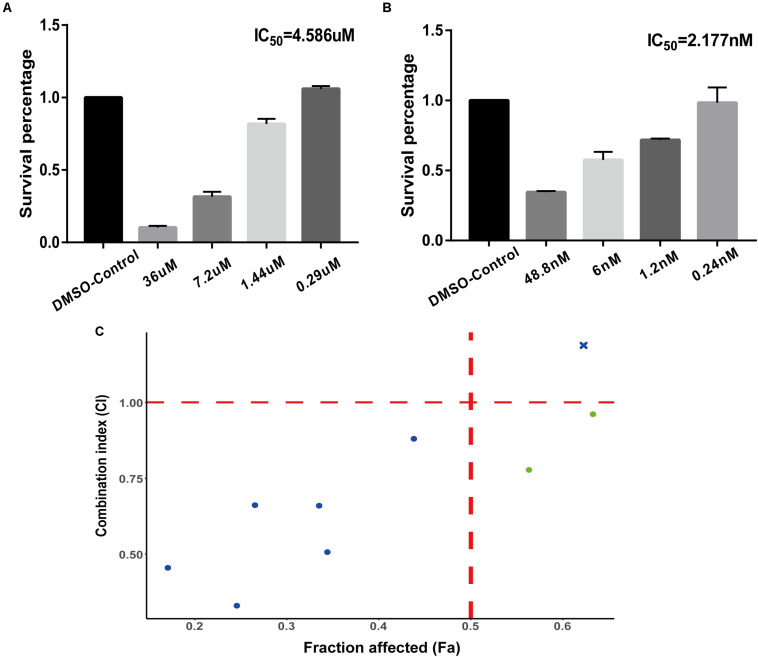
Experiments of drug combinations. **(A)** IC_50_ value of monobenzone against the A375 cell line. **(B)** IC_50_ value of paclitaxel against the A375 cell line. **(C)** The scatter plot of CI versus fraction affected (Fa).

The experimental validation results of the IC_50_ values of the two tested drugs are shown in Figures 3A,B (monobenzone Figure 3A, paclitaxel Figure 3B). Paclitaxel and monobenzone combinations at different concentrations exhibited synergistic effects on A375 cell lines (CI < 1), and antagonism arose with increasing concentrations (Figure 3C). These results indicated that the combination of paclitaxel and monobenzone might be a promising therapy for melanoma cell proliferation.

## Discussion

Predicting drug combinations is an important research topic in drug discovery because it can reduce costly wet experiments and find potential drug combinations in an efficient way. We proposed drug combination prediction models by utilizing multifeature data on drugs, including drug-target information, drug-indication information, drug chemical structure information, gene expression profiles of drugs and module information on drug indications. The ensemble model outperformed the state-of-the-art classification method. The biological experimental results for a predicted drug combination (paclitaxel and monobenzone) validated our ensemble model prediction. We believe our methods are a promising strategy to discover potential drug combinations.

## Data Availability Statement

Publicly available datasets were analyzed in this study. This data can be found here: https://github.com/Ronlee12355/drug_combination_prediction.

## Author Contributions

H-YZ and L-DZ conceived and supervised the study and made manuscript revisions. JL analyzed and visualized the data, and wrote the manuscript. X-YT conducted the biological experiments. All authors contributed to the article and approved the submitted version.

## Conflict of Interest

The authors declare that the research was conducted in the absence of any commercial or financial relationships that could be construed as a potential conflict of interest.

## Footnotes

1 https://www.graphpad.com/

2 www.combosyn.com

## Supplementary Material

FIGURE S1 Results of feasibility analysis. **(A)** Student’s *t*-test results for five features indicating that the values of similarity in drug target information, drug indication information, drug structure information and drug expression information between the positive and negative classes were significant. **(B)** Kolmogorov-Smirnov test results for five features indicating that the distribution of similarity in drug target information, drug indication information, drug structure information and drug expression information between positive and negative classes were significant. **(C)** Model importance of five NRM models in the ensemble classifier.

TABLE S1 Performances of three classification prediction models with different negative sample ratio levels.

TABLE S2 Performances of ensemble models evaluated by 3-, 5- and 10-fold validation.

TABLE S3 Combination index (CI) values of paclitaxel and monobenzone against A375 cancer cells.

TABLE S4 Performances of five neighbor recommender method models.

TABLE S5 Results of comparison with state-of-the-art methods.
