# Supplementary figures and images for: A Machine Learning Method for Drug Combination Prediction
Source: Front Genet. 2020 Aug 25;11:1000. doi: 10.3389/fgene.2020.01000 (PMC7477631; doi:10.3389/fgene.2020.01000)

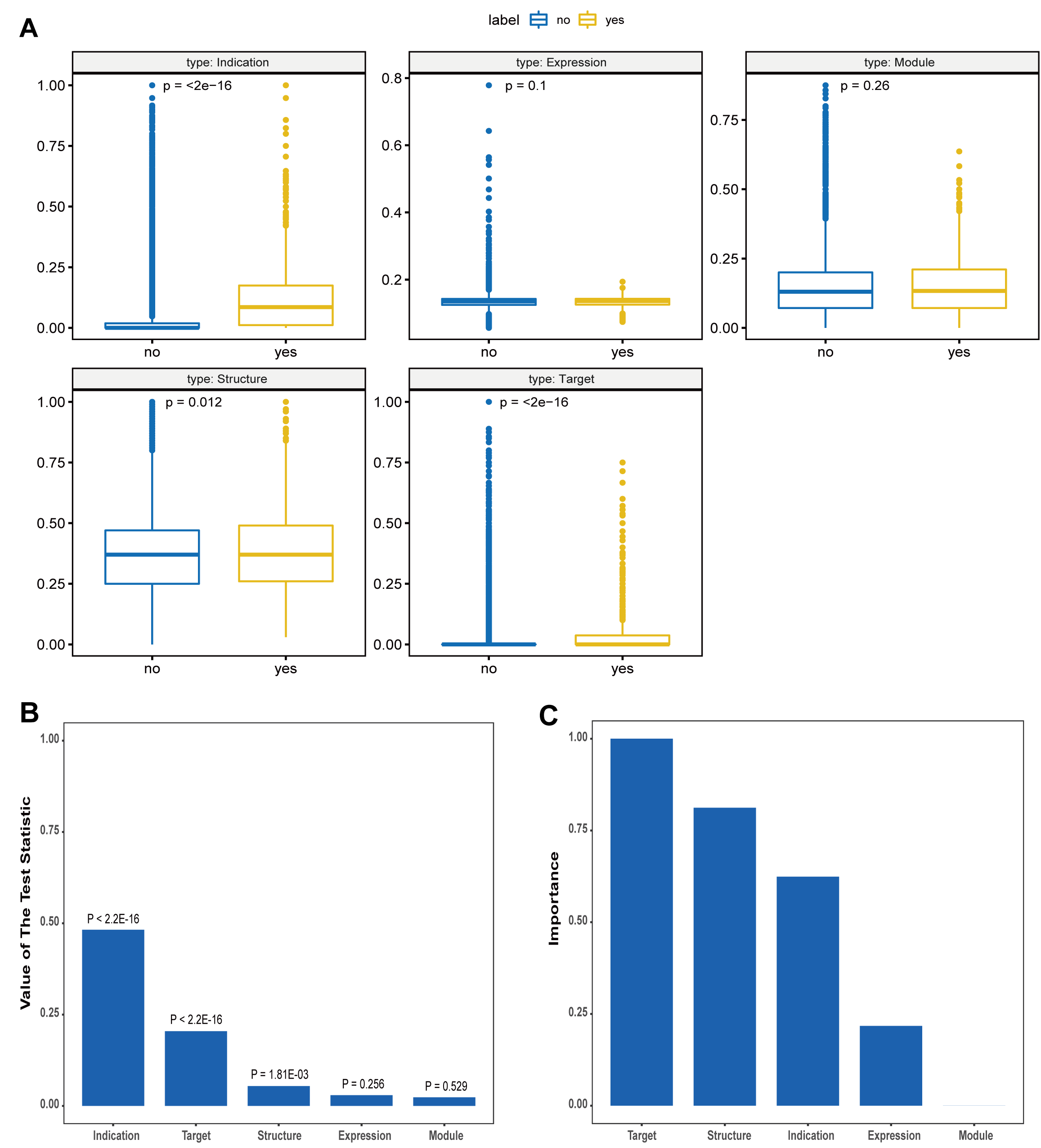

Supplement: FIGURE S1 — Results of feasibility analysis. (A) Student’s t-test results for five features indicating that the values of similarity in drug target information, drug indication information, drug structure information and drug expression information between the positive and negative classes were significant. (B) Kolmogorov-Smirnov test results for five features indicating that the distribution of similarity in drug target information, drug indication information, drug structure information and drug expression information between positive and negative classes were significant. (C) Model importance of five NRM models in the ensemble classifier. [file Image_1.TIF]
